# Disparities in Global Rabies Burden from GBD 2021: Children, older adults, and low-SDI countries at continued risk

**DOI:** 10.1371/journal.pntd.0013630

**Published:** 2025-10-17

**Authors:** Chenxuan Yang, Mei Dang, Longjiang Wu, Gelin Jin, Qinqin Deng, Yihui Chen, Song Jin, Wang Chen, Chenlu Zhang

**Affiliations:** 1 School of Biological Science and Engineering, Shaanxi University of Technology, Hanzhong, Shaanxi, China; 2 National University of Singapore, Singapore, Singapore; 3 Tongji Medical College of Huazhong University of Science and Technology, Wuhan, Hubei, China; 4 Kyrgyz State University named after I. Arabaev, Bishkek, Kyrgyz Republic; London School of Hygiene and Tropical Medicine, UNITED KINGDOM OF GREAT BRITAIN AND NORTHERN IRELAND

## Abstract

**Background:**

Rabies is a fatal yet preventable viral infection of the central nervous system zoonotic disease, with an almost 100% case fatality rate once symptoms appear. Despite the availability of effective vaccines and post-exposure prophylaxis, rabies continues to cause substantial mortality, particularly in parts of Asia and across the African continent where access to post-exposure prophylaxis (PEP), timely diagnosis, and dog vaccination programs remain limited.

**Objectives:**

Rabies is a nearly invariably fatal zoonotic encephalitis caused by lyssaviruses, with an estimated 59 000 human deaths annually, predominantly in Asia and cross the African continent where access to PEP and dog vaccination is limited. Due to historical underreporting and fragmented surveillance, the true burden remains unclear. Using Global Burden of Diseases (GBD) 2021 data, we systematically assess the global, regional, and national burden of rabies from 1990 to 2021 to identify key drivers of change and persistent disparities.

**Methods:**

We extracted rabies incidence and disability-adjusted life-year (DALY) data from the GBD 2021 via the Global Health Data Exchange. Countries were stratified into five sociodemographic index (SDI) categories. Age-standardized rates per 100 000 population were computed using the GBD world standard. We applied Bayesian age-period-cohort (APC) modeling to characterize temporal trends and project future age-standardized incidence rate (ASIR) and age-standardized DALY rates (ASDR), decomposition analysis to partition burden changes into epidemiological, population growth, and aging components, and frontier benchmarking to assess each country’s performance relative to its SDI.

**Results:**

From 1990 to 2021, global rabies ASIR fell by 69.4% (0.42 to 0.13/100 000) and annual cases by 54% (22 035–10 181). Over the same period, DALYs declined 58.4% (1 368 780–569 550) and ASDR dropped from 24.48 to 7.51/100 000. In 2021, the highest ASIRs were in Nepal (1.71), Ethiopia (1.05) and Malawi (0.77/100 000). Age-period-cohort analysis showed net drifts of -3.96%/year for incidence and -3.90%/year for DALYs. Decomposition attributed ~170% of incidence decline to epidemiological gains, offset by ~60% from population growth and ~52% from aging.

**Conclusions:**

Scale up mass dog vaccination, ensure affordable PEP and strategic Pre-exposure prophylaxis, focus on high-risk age cohorts, bolster surveillance and data-driven governance, and sustain multisectoral investment through the phased “Zero by 30” framework. By aligning programmatic efforts with the demonstrated successes in high-performing countries and addressing the implementation deficits in lagging regions, the global community can accelerate progress toward the World Health Organization (WHO) goal of zero dog-mediated human rabies deaths by 2030.

## Author summary

Rabies is a fatal yet preventable viral infection of the central nervous system, with an almost 100% case fatality rate once symptoms appear. Using data from the Global Burden of Disease Study 2021, this study presents a comprehensive assessment of global, regional, and national rabies trends from 1990 to 2021. We estimated incidence and disability-adjusted life-years (DALYs) and applied age-period-cohort (APC) modeling, decomposition analysis, and frontier benchmarking to evaluate temporal patterns, demographic drivers, and inequalities across 204 countries and five sociodemographic index (SDI) levels. Results revealed a substantial global decline in incidence and DALYs over the study period. However, the burden remains disproportionately concentrated in low-SDI regions such as the African continent and South Asia. APC analysis revealed a persistent U-shaped distribution in incidence and burden, with peaks in children aged 5–9 years and adults over 70 years. Decomposition analysis showed that epidemiological improvements, rather than population aging or growth, accounted for most of the reductions in burden. Nonetheless, in regions like Eastern Sub-Saharan Africa and South Asia, demographic pressure partially offset gains. Frontier analysis further exposed persistent inequalities, with countries like Nepal, Ethiopia, and Malawi falling significantly behind their expected performance levels, while high-SDI countries and parts of Latin America sustained near-zero burden since 1990. These findings underscore the urgent need for intensified One Health strategies, equitable post-exposure prophylaxis (PEP) access, and targeted interventions for high-risk groups, especially children and the elderly, to close the remaining gaps and achieve the World Health Organization (WHO) goal of zero human deaths from dog-mediated rabies by 2030.

## 1. Introduction

Rabies is one of the most lethal yet entirely preventable zoonotic diseases, caused by neurotropic viruses of the Lyssavirus genus within the *Rhabdoviridae* family [1–4]. It induces acute, progressive encephalomyelitis in humans and animals, and once clinical symptoms appear, the case fatality rate approaches 100% [1,5]. Despite the availability of highly effective vaccines and life-saving post-exposure prophylaxis (PEP), rabies continues to exact a severe toll, particularly in low-resource settings [6]. Globally, an estimated 59,000 human deaths occur each year, with the vast majority concentrated in parts of Asia and cross the African continent where access to PEP, timely diagnosis, and dog vaccination programs remain limited [7].

The burden of rabies is largely associated with dog-mediated transmission, particularly in children under 15 years of age who account for a disproportionate share of fatalities due to increased exposure risk and barriers to timely treatment [8,9]. Moreover, rabies is deeply entangled with structural inequalities in health access [10], with its continued persistence representing a failure of equitable healthcare delivery, veterinary infrastructure, and disease surveillance systems. Although rabies is entirely vaccine-preventable, these disparities mean that the disease remains a daily reality for populations in resource-limited areas, despite being virtually eliminated from high-income settings [11,12].

Globally, rabies burden has historically been underestimated due to underreporting, misdiagnosis, and limited surveillance systems [13]. This underreporting not only obscures the true scope of the problem but also hampers strategic public health responses and sustainable resource allocation. The Global Burden of Disease (GBD) 2021 study addresses this challenge by providing harmonized, comparable estimates of disease burden, including incidence and disability-adjusted life years (DALYs), across 204 countries and territories over a span of more than three decades [14,15]. GBD estimates are critical for understanding not only the absolute and relative burden of rabies but also for evaluating progress toward global targets, such as the World Health Organization (WHO) goal of “Zero by 30” which aims the elimination of dog-mediated human rabies deaths by 2030 [16–18].

This present study uses GBD 2021 data to conduct a comprehensive analysis of the global, regional, and national burden of rabies from 1990 to 2021 to identify key drivers of change and persistent disparities. By integrating age-period-cohort (APC) modeling, decomposition analysis, and frontier benchmarking, we provide a quantified understanding of temporal dynamics, demographic contributions, and equity gaps in rabies control. APC analysis enables the identification of age- and cohort-specific risk patterns, while decomposition methods quantify the relative contributions of aging, population growth, and epidemiological change to the net burden. Finally, frontier analysis situates each country’s performance relative to its sociodemographic index (SDI), offering a data-driven framework to evaluate efficiency and identify outliers.

This study contributes to a more granular and policy-relevant understanding of rabies epidemiology in the SDG and One Health era. By highlighting where, why, and for whom the burden of rabies remains unacceptably high, it informs future investments, advocacy, and international collaboration required to meet the global rabies elimination targets.

## 2. Methods

### 2.1 Data sources and processing

This study’s data were downloaded via the Global Health Data Exchange (GHDx) platform (http://ghdx.healthdata.org/gbd-results-tool) on May 1, 2025. For gathering the data utilized in the models, the GBD conducts thorough reviews, takes advantage of spontaneous searches, and integrates information supplied by national collaborators and the WHO. Data seeking is iterative and ongoing in process in order to identify new sources. Moreover, SDI data were used to evaluate the influence of socioeconomic factors on disease burden. Countries and territories were grouped into five SDI strata in the GBD 2021, ranging from low to high development. SDI ranges from 0 to 1 (lowest to highest), providing a holistic measure of a region’s socio-demographic status. Depending on their 2021 SDI values, countries were categorized as low SDI (0-0.45), low-middle SDI (0.45-0.61), middle SDI (0.61-0.69), middle-high SDI (0.69-0.81), and high SDI (0.81-1).

This study relied on the robust data from the Global Burden of Disease Study 2021, managed by the Institute for Health Metrics and Evaluation (IHME), to provide a detailed analysis of rabies [19]. The dataset encompassed a wide array of metrics, including the incidence, prevalence, mortality, and DALYs for these diseases across 204 countries [20]. Our data extraction process has honed in on both the absolute and age-standardized rates of incidence and prevalence, as well as the mortality rates and disability-adjusted life years (DALYs) associated with rabies. Building on these data, we aimed to assess global, regional, and national rabies trends from 1990 to 2021, disentangle the drivers of change, and highlight persistent disparities.

### 2.2 Disease definition

Human rabies cases were defined in line with WHO clinical and laboratory criteria (e.g., acute encephalitis in a bitten patient, confirmed by viral detection or antigen/antibody assays) [4]. Human rabies is a vaccine-preventable, zoonotic viral disease characterized by an almost invariably fatal, progressive encephalomyelitis caused by lyssaviruses of the family *Rhabdoviridae*, most commonly transmitted through the bite or scratch of an infected mammal, particularly dogs in endemic regions, following an incubation period that typically ranges from days to months, with definitive diagnosis based on laboratory confirmation (e.g., fluorescent antibody testing and serology) complemented by clinical presentation and epidemiological exposure history.

### 2.3 Estimation of incidence and DALYs

Overall, we included incidence and DALYs for rabies. Age-standardized rates (ASR) per 100 000 people were estimated using the GBD world population age standard [21]. Change in incidence and burden across time was estimated by comparing the change in age-standardized rate and the change in total numbers. The GBD 2021 geographical hierarchy included 204 countries and territories aggregated into 21 regions and seven super-regions. DALYs were estimated at all levels of this geographical hierarchy, by sex, for different age groups (age 0–95 years and older), and for every year from 1990 to 2021. We estimated 95% uncertainty intervals (UIs) for all estimates. Microsoft Excel, Microsoft PowerPoint, and R studio (version 4.0.5) were used to generate all tables and figures.

### 2.4 Statistical analysis

We initiated our analysis with descriptive statistics to summarize the central tendencies and dispersion of the data. This approach provided an overview of the disease burden across different geographies, age groups, and time periods. Descriptive statistics, including measures of incidence, prevalence and DALYs, were computed for age-standardized rates, and stratified by sex and age group. Age groups were stratified into 5-year intervals (e.g., under 5, 5–9, etc.), consistent with GBD protocols. Given the inherent variability in data collection and modeling, we performed uncertainty analysis to quantify the level of uncertainty in our estimates. We calculated 95% uncertainty intervals (UIs) for all our estimates to account for potential errors and variability. Statistical analysis were executed using R software (version 4.0.5) for its robust statistical capabilities ggplot2 package for complex data manipulations and visualizations.

### 2.5 Modeling approaches

APC modeling was applied to investigate temporal patterns in rabies burden using population-based count and person-year data, with birth cohort defined as period minus age [22]. This framework simultaneously estimates the influence of age, calendar period, and birth cohort, yielding several epidemiologically meaningful parameters [23]. Age effects are reflected in longitudinal and cross-sectional age curves, while period effects capture temporal trends that influence all age groups and are expressed as fitted trends and period rate ratios (RRs) relative to a reference period. Cohort effects represent generational differences across groups born in the same years and are summarized by cohort RRs relative to a reference cohort, as well as local drifts that indicate age-specific estimated annual percentage changes (EAPCs). In addition, the model provides net drift, which represents the overall log-linear trend in age-adjusted rates across periods and cohorts. All analyses were conducted using the National Cancer Institute’s APC Web Tool (https://analysistools.cancer.gov/apc/) and R software (version 4.0.5), with full reproducibility ensured through freely available code, which can be obtained directly from the tool’s website.

Frontier benchmarking was applied within the GBD framework to evaluate inequalities in rabies burden in relation to the SDI. The frontier was defined as the lowest level of incidence or DALY rates observed at a given SDI, representing the best attainable performance [24]. The frontier distance (efficiency gap) was calculated as the difference between a country’s observed burden and the frontier estimate for the same SDI, with larger gaps indicating lower efficiency. To further quantify disparities, a relative efficiency index was derived to standardize country performance against the frontier, where values closer to 1 denoted higher efficiency. In addition, subgroup analyses were performed by SDI level (low, middle, high) and by geographic region to examine variations in frontier efficiency across different development contexts.

Decomposition analysis was applied to disentangle the drivers of changes in the absolute number of age-related disease burdens [25]. This approach allowed us to quantify the extent to which differences in underlying factors contribute additively to variations in overall disease burden between groups. In this study, we decomposed changes in incidence and DALYs from 1990 to 2021 into three components: aging, epidemiological change, and population, thus estimating the relative contribution of each factor to the observed trends.

### 2.6 Ethics statement

The data utilized in this study were obtained from publicly accessible GBD data and no personal information and privacy were involved. All the information about ethical standards is available through the official website (http://www.healthdata.org/gbd). Consequently, ethical approval and informed consent were not applicable.

## 3. Results

### 3.1 Global incidence and DALYs of rabies in 2021

**Fig 1:**
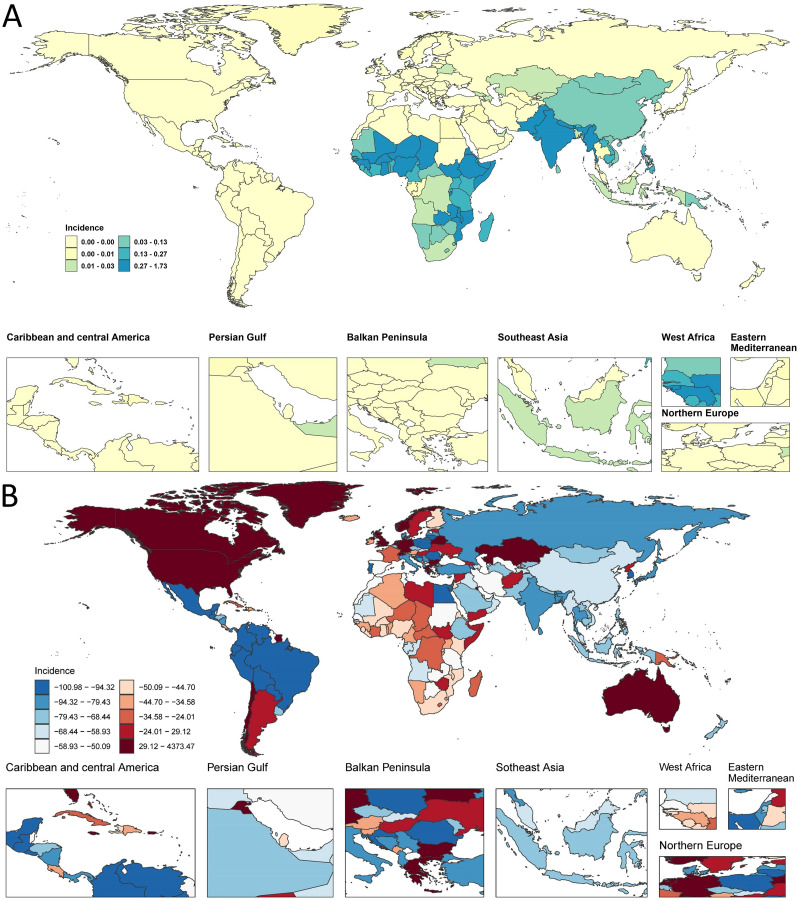
Global distribution and temporal trends in age-standardized incidence rates of rabies from 1990 to 2021. **(A)** Age-standardized incidence rates of rabies per 100,000 population in 2021. **(B)** Percentage change in rabies age-standardized incidence rates between 1990 to 2021. Negative values indicate reductions in incidence, while positive values indicate increased trends. Subregional insets provide detailed visualization for selected regions. Note: The basemap shapefile was from R package ‘rnaturalearth’ version 1.1.0 in the CRAN package repository. https://CRAN.R-project.org/package=rnaturalearth.

In 2021, the age-standardized incidence rate (ASIR) of rabies exhibited pronounced global variation (Fig 1A), with the highest rates reported in Federal Democratic Republic of Nepal (1.71 per 100,000 population), Federal Democratic Republic of Ethiopia (1.05 per 100,000 population), Republic of Malawi (0.77 per 100,000 population), Federal Republic of Somalia (0.63 per 100,000 population), and Republic of the Niger (0.47 per 100,000 population). Several other countries, including Mongolia (0.05 per 100,000 population), Solomon Islands (0.03 per 100,000 population), and Central African Republic (0.03 per 100,000 population), also reported moderate incidence rates. These countries are primarily located in sub-Saharan Africa and Southeast Asia, where dog-mediated rabies remains endemic and access to preventive interventions is limited. In contrast, most high-income countries and well-resourced health systems recorded near-zero ASIRs. For instance, Kingdom of Spain, Republic of Korea, Czech Republic, Republic of Nicaragua, and Republic of Honduras reported negligible rates, suggesting long-standing interruption of transmission and effective disease surveillance.

**Fig 2:**
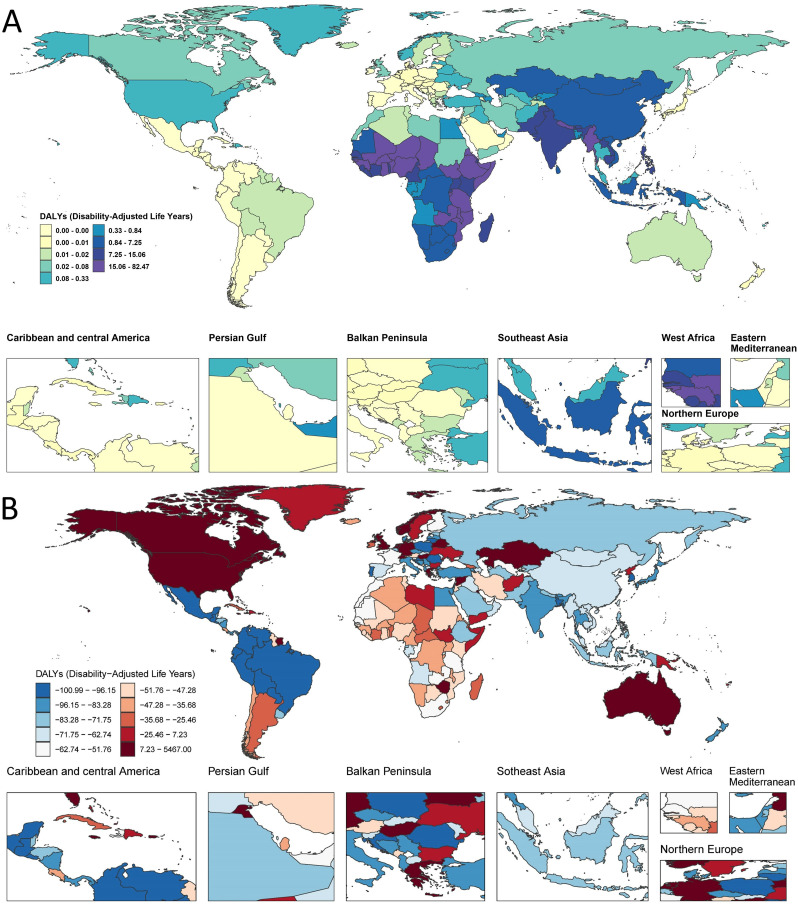
Global distribution and temporal trends in rabies burden measured by ASDR from 1990 to 2021. **(A)** DALY ASRs per 100,000 population attributable to rabies in 2021. **(B)** Percentage change in DALYs ASR between 1990 to 2021. Negative values indicate reductions in rabies burden, while positive values denote increasing trends. Sub-regional insets are provided for areas of interest including the Caribbean, Persian Gulf, Balkan Peninsula, Southeast Asia, West Africa, Eastern Mediterranean, and Northern Europe. Note: The basemap shapefile was from R package ‘rnaturalearth’ version 1.1.0 in the CRAN package repository. https://CRAN.R-project.org/package=rnaturalearth.

Correspondingly, the age-standardized DALY rates (ASDR) also showed geographic clustering in 2021 (Fig 2A and Table 1). The greatest rabies-related DALY burdens were observed in Federal Democratic Republic of Nepal (81.66 per 100,000), Federal Democratic Republic of Ethiopia (58.03 per 100,000), and Federal Republic of Somalia (31.78 per 100,000), reflecting high fatality rates due to delayed or absent post-exposure prophylaxis (PEP). Other African and South Asian countries, such as Republic of Niger (26.73 per 100,000), Republic of Malawi (25.45 per 100,000), and Republic of Mozambique (23.80 per 100,000), also featured prominently among high-burden regions. In contrast, Czech Republic, Republic of Croatia, Kingdom of Spain, Portuguese Republic, and Republic of Korea were among the lowest, highlighting their success in rabies control and elimination.

**Table 1:** The number of incidence, DALY and ASDR of rabies in 1990 and 2021.

	1990			2021		
Characteristics	Incidence Number (95% UI)	DALY Number (95% UI)	ASDR per 100,000 (95% UI)	Incidence Number (95% UI)	DALY Number (95% UI)	ASDR per 100,000 (95% UI)
**Global**						
**Global**	22035.15(15731.80,28729.20)	1368780.43(978542.27,1786736.75)	24.48(16.30,34.02)	10180.67(6080.47,14293.43)	569550.28(323361.74,828522.16)	7.51(3.88,11.69)
**SDI Regions**						
**High SDI**	10.72(7.34,13.95)	529.50(358.45,701.88)	0.07(0.04,0.11)	17.15(12.39,22.61)	699.56(522.71,914.26)	0.08(0.06,0.11)
**High-middle SDI**	353.77(217.69,479.06)	21212.48(13116.15,28991.81)	2.12(1.10,3.41)	244.26(136.48,364.56)	10360.06(5839.22,15254.71)	0.80(0.37,1.46)
**Middle SDI**	3592.18(2636.35,4417.74)	219108.49(165879.07,266668.41)	12.33(8.30,16.68)	1903.98(1134.38,2794.94)	89043.57(51202.69,131920.34)	3.69(1.95,5.90)
**Low-middle SDI**	11413.97(8211.19,14549.77)	691063.95(495899.78,899196.06)	55.92(35.28,78.83)	3816.81(2453.04,5398.63)	199814.84(122514.01,284866.02)	10.44(5.57,17.17)
**Low SDI**	6660.40(4226.91,10636.52)	436598.55(280848.47,690531.54)	75.87(41.56,133.26)	4195.75(2074.40,7177.38)	269468.40(125691.24,465240.98)	21.88(9.71,40.55)
**Southeast Asia, east Asia, and Oceania**						
**East Asia**	1103.41(647.46,1526.19)	69478.83(40696.73,98020.93)	5.99(3.15,9.21)	626.65(334.70,950.14)	26591.12(14377.31,39906.11)	1.81(0.87,3.03)
**Southeast Asia**	1469.39(1095.44,1967.70)	95996.78(74308.74,130899.54)	18.64(10.27,33.78)	663.38(393.04,972.09)	36335.52(21329.06,54988.19)	5.38(2.62,10.54)
**Oceania**	0.69(0.20,2.24)	17.47(4.76,50.20)	0.65(0.10,2.54)	1.56(0.41,4.45)	48.91(11.46,138.89)	0.57(0.08,2.13)
**Central Europe, eastern Europe, and central Asia**						
**Central Asia**	13.36(7.36,25.21)	869.17(447.18,1745.50)	1.16(0.51,2.61)	9.14(5.91,13.73)	508.47(303.71,844.10)	0.53(0.27,1.02)
**Central Europe**	4.10(3.54,4.85)	150.17(130.22,178.65)	0.12(0.08,0.20)	0.15(0.11,0.19)	2.68(2.01,3.56)	0.00(0.00,0.00)
**Eastern Europe**	11.93(10.81,13.29)	628.26(581.78,680.94)	0.30(0.25,0.34)	5.24(4.21,6.62)	178.98(142.97,225.01)	0.08(0.06,0.12)
**High income region**						
**High-income Asia Pacific**	1.03(0.86,1.19)	44.80(39.18,49.38)	0.04(0.03,0.04)	0.38(0.28,0.46)	5.11(4.18,5.98)	0.00(0.00,0.00)
**Australasia**	0.04(0.03,0.04)	1.94(1.58,2.39)	0.01(0.01,0.02)	0.08(0.06,0.11)	4.03(2.61,6.35)	0.02(0.01,0.03)
**Western Europe**	0.54(0.46,0.60)	18.20(15.35,20.38)	0.01(0.00,0.01)	2.06(1.65,2.48)	47.16(40.29,53.20)	0.01(0.01,0.02)
**High-income North America**	1.64(1.54,1.75)	106.72(100.08,113.44)	0.05(0.04,0.05)	6.57(5.76,7.40)	312.12(278.70,350.39)	0.12(0.10,0.14)
**Latin America and Caribbean**						
**Southern Latin America**	0.01(0.01,0.01)	0.59(0.50,0.70)	0.00(0.00,0.00)	0.02(0.02,0.02)	0.41(0.35,0.49)	0.00(0.00,0.00)
**Caribbean**	0.43(0.24,0.75)	14.06(8.70,20.31)	0.04(0.02,0.09)	0.65(0.32,1.25)	18.35(6.94,37.60)	0.04(0.01,0.12)
**Andean Latin America**	16.80(13.02,20.41)	1060.10(810.35,1315.72)	2.56(1.55,3.95)	0.08(0.04,0.16)	2.11(0.86,3.85)	0.00(0.00,0.01)
**Central Latin America**	65.08(59.51,70.74)	4345.02(3927.21,4781.05)	2.32(1.97,2.74)	0.11(0.09,0.13)	3.95(3.25,4.97)	0.00(0.00,0.00)
**Tropical Latin America**	77.86(55.20,104.74)	5295.30(3611.35,7362.39)	3.12(1.98,4.98)	0.40(0.35,0.46)	19.86(16.93,23.60)	0.01(0.01,0.01)
**North Africa and Middle East**						
**North Africa and Middle East**	93.69(48.26,139.94)	6374.97(3138.94,9863.16)	1.58(0.50,3.41)	15.25(7.28,23.42)	847.39(393.30,1345.83)	0.14(0.04,0.29)
**South Asia**						
**South Asia**	15416.62(11333.56,19204.64)	928413.08(683556.56,1177412.37)	79.86(53.04,110.67)	5188.67(3452.19,7083.06)	257449.30(166294.78,365123.33)	14.18(8.20,22.17)
**Sub-Saharan Africa**						
**Central Sub-Saharan Africa**	16.57(3.47,50.92)	1119.40(258.13,3559.08)	1.72(0.28,5.74)	21.61(4.27,60.09)	1353.96(272.33,4062.37)	0.92(0.14,3.10)
**Eastern Sub-Saharan Africa**	2478.32(1161.58,5343.51)	165399.57(77795.90,348595.50)	76.10(29.63,187.18)	1915.69(739.81,4223.91)	124148.26(46424.88,272056.14)	26.65(9.16,61.42)
**Southern Sub-Saharan Africa**	28.92(16.10,44.39)	1975.24(1141.10,3024.95)	3.23(1.45,6.28)	29.49(17.22,46.51)	1847.47(1101.19,3052.96)	2.24(1.04,4.56)
**Western Sub-Saharan Africa**	1234.73(567.02,2161.81)	87470.75(42004.84,146987.01)	35.31(14.46,67.70)	1693.49(673.58,2704.54)	119825.13(47579.08,192226.93)	19.68(7.41,36.64)

### 3.2 Temporal trends in incidence and DALYs (1990–2021)

Between 1990 and 2021, substantial global progress was observed in reducing both the incidence and overall burden of rabies. Globally, the ASIR declined by 69.4% (from 0.42 to 0.13 per 100,000 population), with remarkable improvements noted in Ecuador (−99.98%), Mexico (−99.93%), Guatemala (−99.84%), Mauritius (−99.76%), and Romania (−99.76%) (Fig 1B). These countries successfully reduced transmission through sustained dog vaccination programs, PEP availability, and surveillance integration across human and veterinary sectors.

Similarly, DALY ASRs showed major reductions during the same period, with Republic of Ecuador (−99.99%), United Mexican State (−99.97%), Republic of Guatemala (−99.86%), Republic of Colombia (−99.83%), and Romania (−99.80%) achieving the largest percentage decreases (Fig 2B). These improvements reflect the synergistic effects of long-term public health investments in rabies prevention. However, a subset of countries showed significant increases in incidence or burden. Notably, Jamaica (4330.17%), Canada (2977.26%), and Trinidad and Tobago (2920.29%) recorded sharp increases in ASIR, despite low absolute rates. Likewise, Canada (5412.87%), Jamaica (1372.86%), and Trinidad and Tobago (1165.24%) reported the highest increases in DALYs ASR (Fig 2B). These rises may reflect improved detection, wildlife spillover, or importation of cases, and highlight the importance of continued vigilance.

### 3.3 Trends in rabies incidence and DALYs across SDI regions (1990–2021)

**Fig 3:**
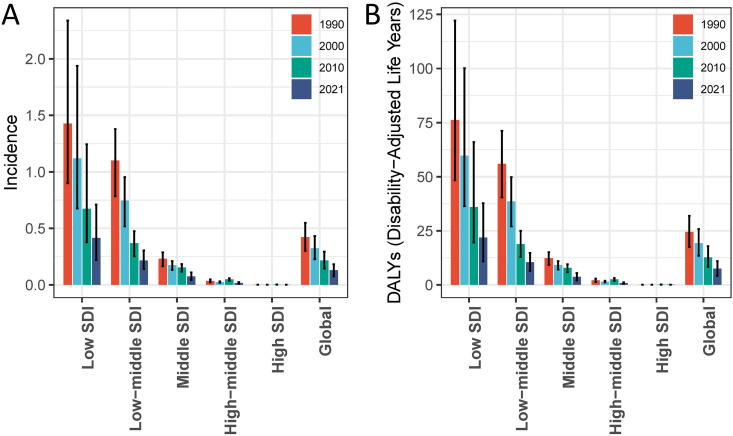
Trends in age-standardized incidence and DALY rates of rabies by SDI region, 1990-2021. **(A)** Age-standardized incidence rates (ASRs) of rabies per 100,000 population across five SDI regions from 1990 to 2021. **(B)** DALY ASR per 100,000 population from 1990 to 2021 across SDI regions. Error bars represent 95% uncertainty intervals derived from the Global Burden of Disease 2021 estimates.

In 1990, the ASIR of rabies was highest in Low SDI regions, reaching 1.43 per 100,000 population. This rate steadily declined over the following three decades, falling to 1.12 in 2000, 0.67 in 2010, and 0.42 in 2021, representing an overall 70.9% reduction. Low-middle SDI regions also experienced a sharp decline, with incidence decreasing from 1.10 in 1990 to 0.22 in 2021, corresponding to an 80.3% reduction. In Middle SDI regions, incidence dropped from 0.23 to 0.075, representing a 67.4% reduction. High-middle SDI regions maintained low incidence levels, decreasing from 0.035 to 0.016, a 54.3% reduction. High SDI regions consistently reported negligible incidence, ranging from 0.0013 to 0.0014, reflecting sustained interruption of rabies transmission and therefore no meaningful percentage reduction (Fig 3A).

The trends in DALY ASRs mirrored those observed for incidence (Fig 3B). In 1990, Low SDI regions bore the highest burden, with a DALY ASR of 76.2 per 100,000 population. This burden declined to 59.8 in 2000, 36.0 in 2010, and 21.9 in 2021, amounting to a 71.3% reduction overall. Low-middle SDI regions showed a comparable decrease, with DALY ASR falling from 55.9 in 1990 to 10.4 in 2021, an 81.4% reduction. Middle SDI regions saw DALY ASR decline from 12.3 to 3.7, representing a 69.9% reduction. High-middle SDI regions declined from 2.11 to 0.80, a 62.1% reduction. High SDI regions maintained a minimal and largely stable burden, changing only slightly from 0.07 in 1990 to 0.08 in 2021. This small percentage change reflects fluctuations around a near-zero baseline and is likely attributable to improved detection rather than a true deterioration in rabies control.

These declines reflect gradual improvements in rabies prevention and control across SDI regions, particularly in access to dog vaccination and post-exposure prophylaxis (PEP). However, incidence and burden in Low and Low-middle SDI regions remained substantially higher than those in middle- and high-SDI regions throughout the study period. This persistent disparity highlights ongoing challenges in achieving equitable access to effective rabies control measures and strengthening health system capacity in resource-limited settings.

### 3.4 Age- and sex-specific trends in rabies incidence and burden (1990 and 2021)

**Fig 4:**
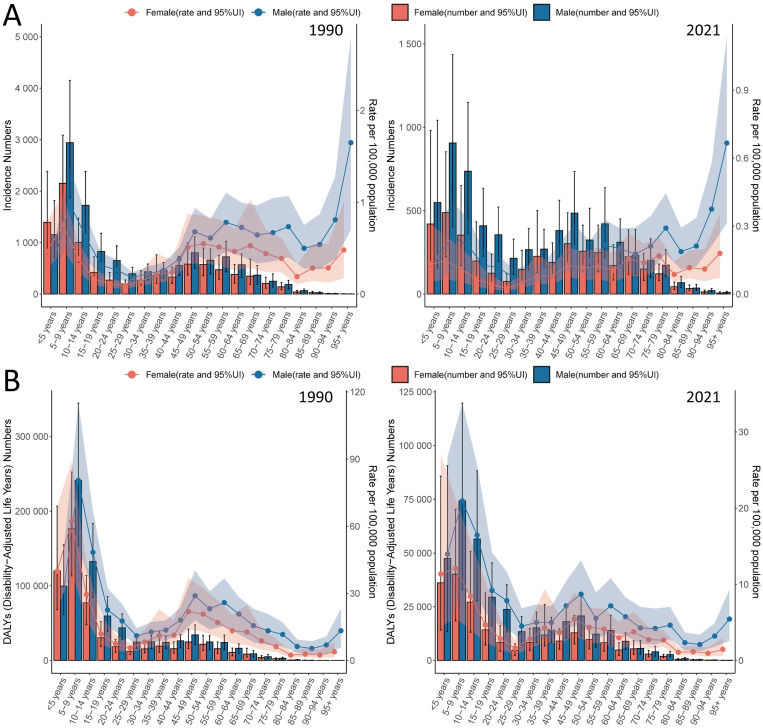
Age- and sex-specific rabies incidence and DALY by sex in 1990 and 2021. **(A)** Age- and sex-specific incidence of dog-mediated rabies in 1990 (left) and 2021 (right): red and blue bars show the number of new cases in each 5-year age group for females and males (with 95% uncertainty intervals), while the corresponding-colored lines and shaded ribbons indicate incidence rates per 100 000 population (95% UI). **(B)** The same structure for disability-adjusted life years (DALYs), with bars representing total DALYs and lines showing DALY rates per 100 000 population. Age groups range from <5 years through ≥95 years.

In 1990, the highest incident cases of rabies occurred among children aged 5–9 years (Fig 4A left). Incident case numbers declined steadily through adolescence and early adulthood. A minor uptick in case numbers was observed around 45–49 years, although overall numbers remained much lower than those in childhood. Beyond 50 years of age, incident case numbers remained consistently low across older age groups. In terms of ASIR per 100,000 population, rabies exhibited a characteristic pattern across the lifespan (Fig 4A). After peaking in children aged 5–9 years (0.98 per 100,000 for males, 0.76 for females), incidence rates declined through adolescence and early adulthood, reaching a low point in the 25–29 years group (0.18 for males, 0.09 for females). Minor fluctuations were observed later, with small upticks after 30 years. From 80 years onward, incidence rates rose progressively, culminating in the highest elderly rates among individuals aged 95 + years and older (1.65 for males, 0.48 for females). However, despite these increases in incidence rates among the elderly, absolute case numbers remained very low. By 2021, rabies incidence rates had decreased substantially across all age groups (Fig 4A right). Among children aged 5–9 years, incidence rates declined from 0.98 to 0.26 per 100,000 in males, and from 0.76 to 0.15 per 100,000 in females, corresponding to reductions of approximately 73%-80%. Similar downward trends were observed across adolescence, adulthood, and older age groups.

The burden of rabies, measured by age-standardize DALYs per 100,000 population, mirrored the trends observed in incidence rates but with sharper gradients (Fig 4B). In 1990, DALY rates peaked among children aged 5–9 years, reaching 80.5 per 100,000 for males and 62.3 for females. After this peak, DALY rates declined sharply during adolescence and early adulthood, reaching a local low at 25–29 years (11.0 for males, 5.6 for females). Between 40–44 and 45–49 years as well as between 50–54 and 55–59 years, a slight increase occurred. Despite these increases, absolute DALY counts among the elderly remained low compared to childhood peaks.

By 2021, rabies DALY rates had declined markedly across all age groups (Fig 4B). Among children aged 5–9 years, DALY rates decreased from 80.5 to 20.9per 100,000 in males and from 62.3 to 12.1 per 100,000 in females, representing reductions exceeding 74%-80%. Modest reductions were also observed among older adults, although a residual burden persisted. Rabies DALY rates closely paralleled incidence patterns, with a peak observed among young children and minor resurgences noted during midlife and older adulthood. Although substantial reductions were achieved across all age groups by 2021, a residual burden persisted among elderly populations. Overall, sex differences in rabies incidence and burden narrowed over time, particularly among adults and older individuals, whereas sex disparities remained relatively stable among young children.

### 3.5 Age-period-cohort (APC) analysis of rabies incidence (1990–2021)

**Fig 5:**
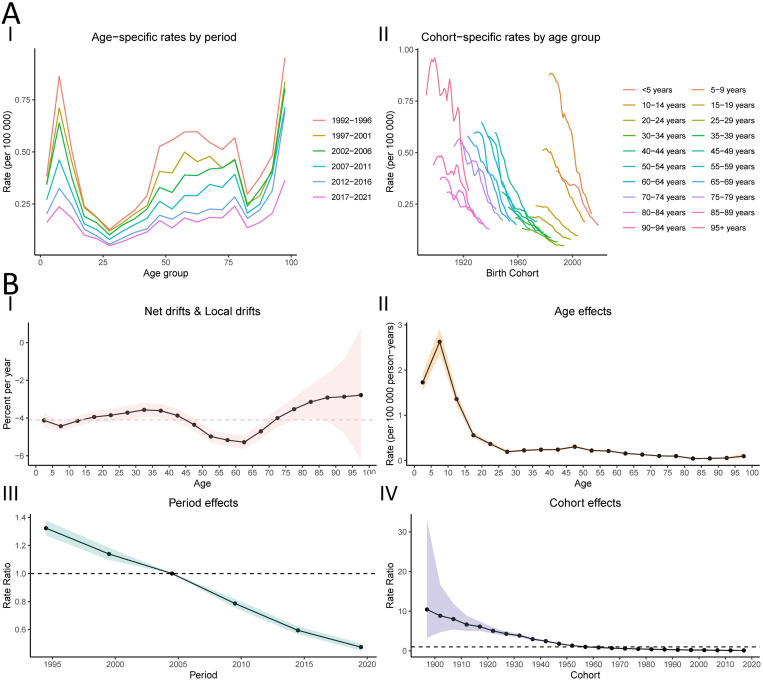
Age-period-cohort analysis of global rabies incidence (1990-2021). **(A)** Observed incidence trends by age group and birth cohort. (I) Age-specific incidence rates across six calendar periods (1992-2021). (II) Birth cohort-specific incidence rates, stratified by age. **(B)** APC model-estimated effects and drift. (I) Net drift and local drifts by age. (II) Age effect adjusted for period and cohort. (III) Period effect showing steady decline in rate ratio from 1.32 in 1995 to 0.47 in 2020. (IV) Cohort effect with RR decreasing from 10.4 in the 1897 birth cohort to 0.10 in cohorts born after 2010.

Age-specific incidence rates across six calendar periods from 1992 to 2021 revealed a consistent U-shaped pattern (Fig 5A-I). In each period, incidence peaked during early childhood and older adulthood. Among children between 5–9 years of age, rates declined from approximately 0.87 per 100,000 in 1992–1996 to 0.24 in 2017–2021 (Fig 5A-I). The lowest incidence was consistently observed between ages 20 and 40, while rates increased steadily after age 80, reaching their highest levels in those aged 95 and older. Importantly, the entire curve shifted downward over time, indicating sustained reductions in rabies incidence across all age groups.

Cohort-specific incidence trends, stratified by age, further underscored generational differences in risk (Fig 5A-II). Individuals born after 1990 experienced uniformly low rates throughout the life course. In contrast, those born before 1950 faced significantly elevated risks, especially during childhood and late adulthood. The most notable improvements occurred among cohorts born between 1960 and 1990, reflecting long-term benefits from expanded dog vaccination campaigns and increased access to post-exposure prophylaxis (PEP).

The estimated net drift in rabies incidence was -3.96% per year, highlighting a sustained annual decline throughout the study period (Fig 5B-I). Local drifts were negative across all ages, indicating declines in rabies incidence at every life stage. The most pronounced reductions occurred in older adults aged 58–63 years, with rates declining by approximately -5.3% per year. Young children under 10 years also experienced steep declines (up to -4.4%). Adults aged 20–45 years exhibited consistently negative local drifts (-3.8% to -3.9%), though the rates of decline were somewhat less extreme.

After adjusting for period and cohort effects, the modeled age-specific incidence peaked at 5–9 years (2.63 per 100,000 person-years), followed by a steep decline through adolescence and early adulthood (Fig 5B-II). Rates remained low during middle age and rose again after age 70, echoing the U-shaped pattern observed in raw data. Period effects demonstrated a clear and consistent downward trend, with rate ratio declining from 1.32 in 1995 to 0.47 in 2020 (Fig 5B-III). This temporal pattern suggests sustained progress in rabies control independent of demographic structure.

Finally, cohort effects revealed a dramatic generational shift in risk (Fig 5B-IV). The rate ratio declined from 10.4 among those born around 1897 to 0.10 among cohorts born after 2010, reflecting a near 100-fold reduction across generations.

### 3.6 Age-period-cohort (APC) analysis of rabies burden (1990–2021)

**Fig 6:**
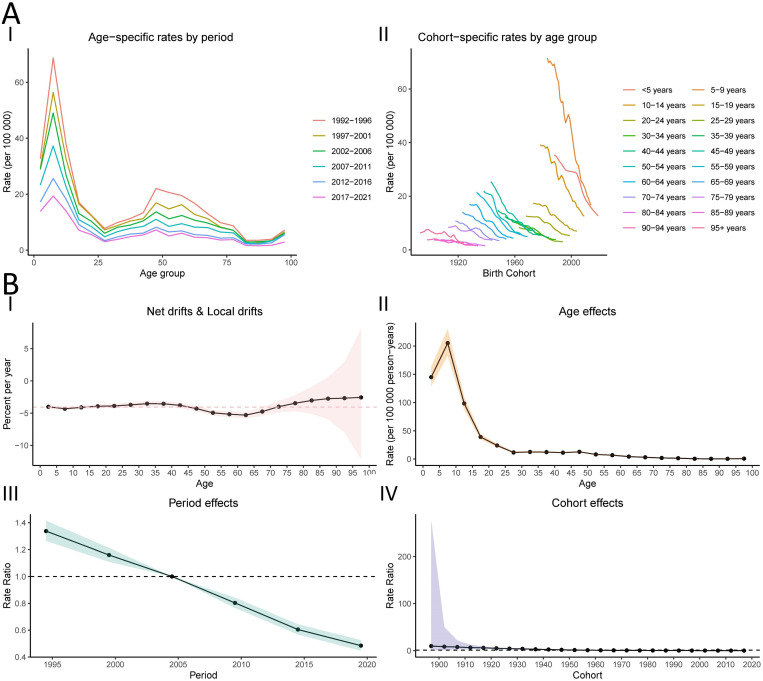
Age-period-cohort (APC) analysis of global rabies burden (1990-2021). **(A)** Observed trends in age- and cohort-specific DALY rates. (I) Age-specific DALY rates per 100,000 population across six calendar periods (1992-1996 to 2017-2021). (II) Cohort-specific DALY rates by age group. **(B)** APC model-based decomposition of temporal effects on DALY rates. (I) Net drift (dashed line) and local drifts (points and 95% CI bands) indicate average annual percentage change across ages. (II) Modeled age effect after adjusting for period and cohort. (III) Period effects reflect temporal risk changes independent of age and cohort. (IV) Cohort effects show a dramatic generational decline in DALY burden.

Across six calendar periods from 1992 to 2021, age-specific DALY rates (Fig 6A-I) exhibited a left-skewed U-shaped distribution, with the highest burden observed in early childhood and a secondary rise in late adulthood. The highest DALY rates were recorded among children aged 5–9 years, reaching 68.9 per 100,000 in 1992–1996 and falling to 19.4 per 100,000 in 2017–2021. Following the childhood peak, DALY rates declined through adolescence and middle adulthood, and rose again in older age. Notably, DALY rates also exhibited a modest midlife rebound (ages 45–60), which was not prominent in incidence patterns. The highest burden remained among children aged 5–9 years throughout all periods. Notably, the entire curve shifted downward over time, reflecting a broad-based decline in burden across age groups and periods.

Cohort analysis indicated a steep and sustained decline in RR across successive generations (Fig 6A-II). The RR peaked at 9.72 among those born in 1897, followed by a gradual and sustained decline across successive cohorts. These trends align with the global expansion of canine vaccination, improved access to PEP, and the strengthening of One Health strategies. Drift analysis (Fig 6B-I) revealed a net drift of -3.90% per year, reflecting a sustained average annual decline in rabies-related DALY rates from 1990 to 2021. Local drifts were negative across all ages, with the sharpest declines in adults aged 55–65 years. In contrast, declines were shallower or statistically uncertain in the very elderly (≥85 years), indicating the need for reinforced protection in advanced age. The modeled age effect showed that the highest DALY rate occurred at age 7.5 years, reaching 205.3 per 100,000 person-years (Fig 6B-II). A steep decline followed through adolescence and early adulthood. From age 30–75, rates remained consistently below 15 per 100,000, before falling further below 1.0 per 100,000 beyond age 80. The lowest DALY rate was observed at age 87.5 years (0.47 per 100,000), after which a minor rebound occurred in centenarians (97.5 years: 0.79 per 100,000), possibly reflecting residual risk among long-surviving vulnerable populations.

Period effects confirmed steady reductions in RR over time, decreasing from 1.34 in 1995 to 0.48 in 2020 (Fig 6B-III). Similarly, cohort effects revealed a dramatic generational shift (Fig 6B-IV). The RR decreased from 9.72 among those born in 1897 to near 0.10 among cohorts born after 2010. This trend underscores the long-term impact of sustained rabies control programs, particularly in endemic regions.

### 3.7 Decomposition analysis of changes in rabies incidence and DALY rates (1990–2021)

**Fig 7:**
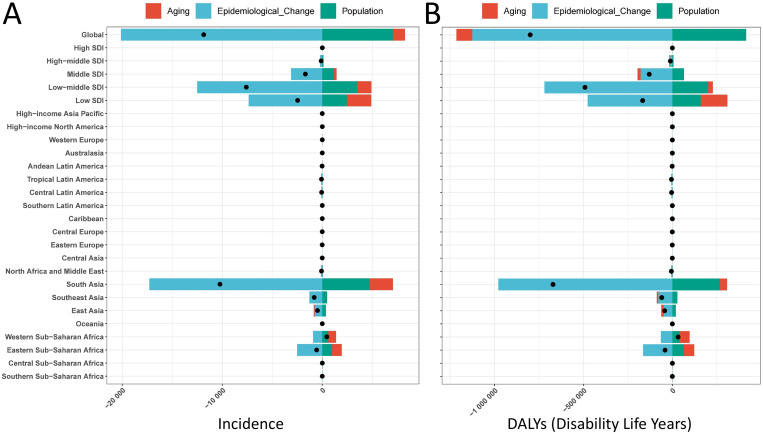
Decomposition of Changes in Rabies Incidence and DALYs by Region (1990-2021). **(A)** Absolute change in rabies incidence attributed to demographic aging (red), population growth (green), and epidemiological change (blue). **(B)** Absolute change in DALY attributed to the same three components. Black dots represent the net overall change in each region. Positive values indicate increased burden, while negative values indicate reductions.

To better understand the driving forces behind the decline in rabies burden, we performed a decomposition analysis of incidence and DALYs, partitioning the net changes into contributions from aging, population growth, and epidemiological change (Fig 7).

Globally, the overall reduction in rabies incidence and DALYs was primarily attributed to favorable epidemiological changes. From 1990 to 2021, 169.8% of the global decline in incidence and 140.7% of the reduction in DALYs were attributable to improved prevention, dog vaccination coverage, and PEP access. In contrast, population growth exerted upward pressure, offsetting 59.5% of the incidence and 51.9% of the DALY reductions, while population aging had comparatively minor effects (Fig 7A and 7B).

At the regional level, epidemiological change was the dominant contributor to burden reduction across nearly all regions, especially in High-income Asia Pacific, East Asia, and Central Latin America, where age-specific rates decreased despite growing and aging populations. In contrast, Western and Eastern Sub-Saharan Africa experienced opposing dynamics, although these regions achieved large absolute reductions in incidence and DALYs, population growth and aging offset gains to varying extents. For example, in Eastern Sub-Saharan Africa, epidemiological improvements reduced incidence by 2,510.2 cases, yet population growth added 965.9 new cases, limiting the net reduction.

In Low- and Low-middle SDI regions, the epidemiological contribution remained critical, accounting for over 164.7% of net declines. Notably, in South Asia, epidemiological improvements accounted for 169.0% of the net decline in incidence, offset by 46.2% from population growth and 22.8% from aging, and for 145.8% of the reduction in DALYs, with offsets of 39.8% and 6.1%, respectively. Similarly, in Southeast Asia, epidemiological change contributed 159.6% of the net reduction in incidence (counteracted by population growth [57.0%] and aging [2.6%]), and 132.2% of the decrease in DALYs, with population growth offsetting 47.2% and aging contributing an additional 15.0% to the net decrease. These patterns underscore the power of targeted prevention in rapidly growing populations.

Notably, a few high-SDI settings, including Australasia and High-income North America showed small net increases in incidence or DALYs, despite declining epidemiological rates. This was largely due to aging populations and enhanced surveillance that may detect more cases in previously underreported older age groups or wildlife-exposed populations.

### 3.8 Frontier analysis of the burden of rabies (1990–2021)

**Fig 8:**
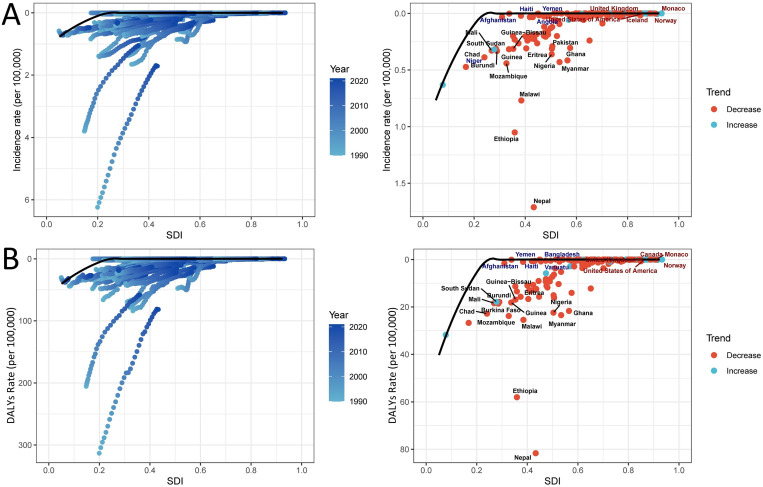
Frontier analysis of age-standardized rabies incidence and DALYs rates by SDI (1990-2021). **(A)** Incidence rate per 100,000 population across SDI spectrum. Left panel: all country-year observations plotted from 1990 to 2021, color-coded by year. Right panel: 2021 values only, with countries labeled and classified by trend direction (increase vs. decrease) relative to 1990. **(B)** DALYs rate per 100,000 population across SDI spectrum. Left panel: all country-year observations from 1990 to 2021, color-coded by year. Right panel: 2021 values with labeled countries and corresponding burden trend direction. The black frontier line represents the best-achieved outcomes (lowest burden) at each SDI level, serving as a benchmark for potential optimization. Countries below the frontier suggest room for improvement relative to their development status.

The frontier analysis revealed substantial variation in national performance relative to socioeconomic development across 1990–2021. Most countries followed a declining trajectory in rabies incidence and DALY rates as their SDI improved (Fig 8A and 8B). However, significant gaps remained between observed burden and the theoretical minimum burden expected for a given SDI level. Countries such as Nepal, Ethiopia, Mozambique, and Malawi showed the largest distances from the frontier line, with consistently elevated incidence and DALY rates over time. These patterns suggest systemic weaknesses in PEP accessibility, dog vaccination coverage, and integrated surveillance. By contrast, a small number of high-SDI countries, such as the United States, Monaco, and Norway showed slight increases in incidence or DALYs from near-zero baselines, though these shifts likely reflect better detection of wildlife rabies rather than failure in human prevention.

## 4. Discussion

This study reveals substantial progress in global rabies control over the past three decades, alongside persistent disparities in incidence and burden across different SDI regions. Although the global ASIR declined by 69.4%, and the DALYs ASR fell from 24.5 to 7.5 per 100,000 population between 1990 and 2021 (S1 Data and Table 1), rabies remains concentrated in resource-limited settings like sub-Saharan Africa and South Asia (Figs 1A and 2A). Despite notable reductions, such as a 70.9% decrease in incidence and a 71.3% decrease in DALYs among Low SDI regions, rabies burden in these areas remained substantially higher than in Middle-, High-middle-, and High-SDI regions. Persistent hotspots in sub-Saharan Africa and South Asia such as Nepal (ASIR 1.71 per 100,000; DALY rate 81.66 per 100,000), Ethiopia (ASIR 1.05; DALY rate 58.03), and Malawi (ASIR 0.77; DALY rate 25.45) highlight multifactorial challenges. Suboptimal dog vaccination coverage, driven by logistical constraints and limited funding, fails to reach the ≥ 70% threshold necessary for herd immunity [26–28]. Uneven and costly access to PEP—averaging US\$108 per course—coupled with frequent stock-outs and centralized distribution, delays life-saving treatment [8,29]. Weak surveillance, centralized reporting systems, and limited community awareness about bite prevention and the urgency of PEP further hinder effective rabies control [30].

In contrast, most high-SDI countries and parts of Latin America have successfully maintained near-zero rabies incidence and burden since 1990. Their success is largely attributed to sustained investments in integrated rabies control programs, which include mass dog vaccination, universal PEP access, effective dog population management, and strong surveillance systems [31,32]. The minimal and stable DALY ASRs in High SDI countries, remaining around 0.07-0.08 per 100,000, illustrate the potential impact of coordinated and well-resourced public health efforts. These experiences offer important models for achieving global rabies elimination.

Middle- and High-middle SDI regions exhibited mixed patterns. Many countries within these groups achieved steady declines in incidence and burden, reflecting gradual improvements in vaccine coverage and health system capacity. For instance, Middle SDI regions saw a 67.4% reduction in incidence and a 70.0% reduction in DALYs between 1990 and 2021. However, DALY ASRs in High-middle SDI regions showed a temporary increase, rising from 1.49 per 100,000 in 2000 to 2.59 per 100,000 in 2010, before declining to 0.80 per 100,000 by 2021 (S1 Data). These transient setbacks may be linked to inconsistent vaccination efforts, expansion of urban areas increasing human-animal interactions, or gaps in the integration between human health and veterinary services.

Encouragingly, several countries demonstrated that rabies burden can be drastically reduced with sustained political commitment and cross-sectoral coordination. Romania, South Korea, and Ecuador achieved reductions exceeding 90% in both incidence and DALYs. Since 2006 the Seoul Metropolitan Government has conducted oral rabies vaccine bait distributions twice yearly; municipal authorities report that these sustained campaigns have contributed to zero human rabies cases since 2005 and zero animal cases since 2014 [33]. Meanwhile, in rural Ecuador the non-governmental organization Amici Cannis has provided free mass dog vaccination for more than ten years, demonstrating how sustained community vaccination can reduce bite risk and strengthen local rabies prevention efforts [34]. Because GBD inputs do not encode programmatic measures directly, we present these links cautiously as plausible explanations rather than causal proofs. Their success demonstrates the feasibility of rabies elimination through One Health strategies, integrating mass dog vaccination, PEP availability, community education, and surveillance systems that span human and animal health domains [35–37].

Although small increases in incidence and burden were observed in a few high-income countries, including Canada, Jamaica, and the United States (Figs 1B and 2B), these changes are minimal in absolute terms and should be interpreted cautiously. These increases, while numerically small, they are epidemiologically important because they highlight persistent risks, particularly wildlife rabies reservoirs, international animal movements, and occasional importations, that can produce detectable upticks even the regions or countries where dog-mediated transmission has been interrupted. These trends may partly reflect enhanced surveillance and diagnostic capacity, but also suggest emerging risks from wildlife reservoirs, increased international animal movement, and growing interspecies contact [38,39]. They underscore the need for continuous vigilance, high-quality surveillance, diagnostic capacity, and rapid response systems in rabies-free or near-elimination settings, rather than indicating a substantive reversal of long-term control gains.

Beyond geographic and socioeconomic disparities, the analysis revealed consistent age- and sex-related patterns in rabies burden (Fig 3). In 1990, incidence and DALYs were highly concentrated among children aged 5–9 years, likely due to behavioral risk and limited access to PEP. Although absolute case numbers remained low among older adults, incidence and burden rates increased after age 70, with additional minor upticks in mid-adulthood (e.g., 45–49 and 55–59 years), possibly driven by declining immunity, comorbidities, and underreporting. By 2021, age-specific rates had declined across all life stages, with particularly sharp reductions among children (Fig 4). Sex differences in rabies burden narrowed over time, particularly among adults and older populations, likely reflecting more uniform access to PEP and public health messaging. However, disparities remained relatively stable among young children, suggesting the need for targeted education and prevention in high-risk pediatric populations.

APC analysis further elucidated the dynamic evolution of rabies burden over the past three decades (Figs 5 and 6). Both incidence and DALY rates exhibited U-shaped age distributions, with the highest burden concentrated in children aged 5–9 years and a secondary rise in older adulthood. The steep declines in early-life incidence (from 0.87 to 0.24 per 100,000) and DALY rates (from 66.6 to 16.7 per 100,000) between 1992–1996 and 2017–2021 reflect the substantial gains from expanded PEP coverage, improved dog vaccination, and community awareness. While incidence remained low during middle age, DALY patterns revealed an additional modest rebound in midlife (ages 45–60), likely driven by the long-term sequelae and health system challenges associated with rabies exposure in older working-age adults.

Cohort-specific trends revealed dramatic generational improvements, with rate ratio declining by approximately 97% for both incidence and DALYs from over 9.7 in the 1897 cohort to 0.10 in those born after 2010. These sustained reductions align with the global rollout of integrated One Health strategies, particularly since the 1960s. Drift analysis underscored these trends, with net annual reductions of -3.96% for incidence and -4.06% for DALYs. Local drifts were uniformly negative, with the most rapid declines in adults aged 57.5-62.5 years (-5.2% to -5.3%) and in children under 10 (-4.3% to -4.4%). While period effects confirmed steady improvements over time (RR dropped from 1.32 to 0.47 for incidence and 1.34 to 0.48 for DALYs), the broader generational shift, spanning over a century, emerged as the most powerful driver of progress. Together, these findings reinforce the dual necessity of continued prevention in children and elderly populations, and sustained structural investment across time and generations.

Decomposition analysis indicated that epidemiological improvements such as better surveillance, expanded vaccination, and scaled-up PEP were the primary drivers of global burden reduction, outweighing the counterbalancing effects of population growth and aging (Fig 7). However, in sub-Saharan Africa and other low-resource settings, demographic pressure diluted these gains. Efficiency frontier analysis reinforced this point, revealing that many countries remain well above expected incidence and DALY rates relative to their SDI level (Fig 8). This highlights large and actionable gaps in implementation efficiency, rather than inevitable limitations due to economic development. Notably, a few countries with modest SDI levels approached the frontier line, suggesting that effective interventions can yield substantial health gains even in lower-resource settings. These findings reaffirm the potential for targeted, context-appropriate strategies to close the implementation gap and accelerate progress toward zero human rabies deaths.

Overall, the combined frontier and decomposition analysis suggest that the majority of global progress against rabies stems from genuine public health advancements, particularly in One Health-driven strategies rather than passive demographic transitions. However, continued demographic pressure especially in low-resource settings necessitate adaptive strategies to sustain momentum and achieve global elimination targets.

Taken together, these findings highlight clear epidemiological priorities: large absolute burdens concentrated in low-SDI settings, pronounced pediatric vulnerability (ages 5–9), steady cohort-level improvements, and measurable implementation inefficiencies relative to SDI-conditioned frontiers. These patterns point to a focused set of evidence-based interventions. In particular, achieving and sustaining ≥70% canine vaccination coverage is key to interrupting transmission [27,40]; dose-sparing intradermal PEP regimens and improved PEP provisioning can reduce costs and shorten delays in care [2,41]; and Integrated Bite Case Management (IBCM) and other One Health surveillance approaches improve detection and cross-sector coordination [42,43]. The recommendations that follow therefore map directly onto the epidemiological signals identified here and are supported by the international “Zero by 30” strategy and the peer-reviewed literature on vaccination thresholds, PEP strategies, and IBCM effectiveness [16,44]. Below we translate these links into six targeted recommendations, each accompanied by brief rationale and supporting evidence to assist policymakers and program planners.

### 4.1 Policy implications and recommendations

These results are aligned with the WHO’s One Health approach and the “Zero by 30” strategic framework [18], which envisions the elimination of dog-mediated human rabies deaths by 2030 through three phases: Start-Up (2018–2020), Scale-Up (2021–2025), and Mop-Up (2026–2030) [45]. Achieving this vision requires into practice, we recommend the following integrated measures:

1. Achieve ≥70% coverage through mobile clinics, public-private partnerships, and community vaccination events. Collaborate with the Global Alliance for Rabies Control (GARC), the World Organization for Animal Health (OIE), and the Food and Agriculture Organization of the United Nations (FAO) for technical support and vaccine procurement [16,46].
2. Optimize PEP delivery by pre-positioning intradermal PEP kits at district health posts, training healthcare workers in intradermal administration, and integrating bite-risk assessment into routine primary care to reduce costs and improve timeliness [41,47]. Pre-position intradermal PEP kits at district health posts to reduce costs by 60–80%. Train healthcare workers in intradermal administration and integrate bite-risk assessments into primary care.
3. Create national and district rabies task forces linking ministries of health, agriculture, and wildlife. Deploy smartphone-based the Integrated Bite Case Management (IBCM) tools for real-time bite and case reporting [43,48].
4. Deliver culturally tailored messaging via schools, radio, and faith-based gatherings. Emphasize wound washing, safe dog handling, and urgent PEP access-especially targeting children and caregivers [49,50].
5. Integrate rabies control into national health and veterinary budgets. Leverage Gavi’s Vaccine Investment Strategy for PEP funding, and bundle rabies programs with other neglected tropical diseases initiatives [51].
6. Implement standardized monitoring frameworks, set frontier- and decomposition-informed efficiency targets, and regularly assess progress. Adjust national strategies in response to demographic shifts and emerging threats, such as wildlife reservoirs and cross-border animal movements.

This study has several limitations that should be acknowledged. The analysis relies on GBD estimates, which, although standardized and comprehensive, depend heavily on modeled data in regions with limited or poor-quality surveillance, vital registration, and laboratory confirmation. In such settings, estimates may be affected by under-ascertainment, potential misclassification, and temporal changes in reporting completeness, all of which can influence both estimated levels and apparent trends. The limitations of the GBD dataset in analyzing rare events should also be explicitly discussed. For instance, in regions with very low event counts, GBD relies on statistical smoothing, redistribution of ill-defined causes, and covariate modeling, which can result in fractional estimates and produce apparent small increases or decreases in incidence or burden. Acknowledging these data limitations enhances the transparency and credibility of the study, while also underscoring the scientific importance of GBD as the most systematic and comparable source currently available for quantifying health burdens across countries and over time.

In addition, the statistical approaches applied here carry inherent methodological constraints. Age-period-cohort modeling is subject to the identifiability problem between age, period, and cohort effects, and although standard parameterizations and derived estimators such as net drift, local drifts, and rate ratios were used, residual uncertainty in attributing changes to specific temporal dimensions remains. Frontier benchmarking is sensitive to the definition of the frontier and the treatment of outliers, and it cannot fully capture contextual or subnational variability in health system performance. Similarly, decomposition analysis depends on the choice of reference structures and may produce additive contributions that require careful interpretation, particularly when opposing demographic and epidemiological forces are at play. Country-level ecological analyses may mask substantial subnational heterogeneity, and programmatic recommendations derived from such data must be interpreted with caution and adapted to local contexts. Despite these caveats, acknowledging these limitations both enhances the transparency and credibility of our work and highlights priority actions to strengthen rabies surveillance and future assessments.

In summary, our study provides a comprehensive portrait of dog-mediated rabies trends from 1990 to 2021. While the remarkable global decline in incidence and burden underscores the success of coordinated public health efforts, persistent hotspots in low-resource settings reveal critical gaps in policy implementation and equity. The convergence of WHO’s policy directives and the article’s empirical findings illuminate a clear path forward: scale up mass dog vaccination, ensure affordable PEP and strategic Pre-exposure prophylaxis, focus on high-risk age cohorts, bolster surveillance and data-driven governance, and sustain multisectoral investment through the phased “Zero by 30” framework. By aligning programmatic efforts with the demonstrated successes in high-performing countries and addressing the implementation deficits in lagging regions, the global community can accelerate progress toward the WHO goal of zero dog-mediated human rabies deaths by 2030.

## Supporting information

S1 Data Dataset underlying study analysis. (XLSX)
